# Neuropathic pain and central sensitization in patients with rheumatoid arthritis attending a tertiary hospital

**DOI:** 10.1186/s42358-025-00487-y

**Published:** 2025-10-31

**Authors:** Maria Nathalia Gabriela Rocha Pontes de Oliveira, Débora de Jesus Sena, Ana Karla Guedes de Melo, Danielle C. Soares Egypto, Luís Fábio Barbosa Botelho, Alessandra de Sousa Braz

**Affiliations:** 1 Center for Medical Sciences, Federal University of Paraiba, João Pessoa, PB Brazil; 2 Lauro Wanderley University Hospital, Federal University of Paraiba, João Pessoa, PB Brazil

**Keywords:** Rheumatoid arthritis, Neuropathic pain, Central sensitization, Fibromyalgia

## Abstract

**Background:**

In rheumatoid arthritis (RA), the persistence of pain despite adequate inflammatory control may indicate the involvement of nociplastic and/or neuropathic pain (NP).

**Objective:**

To investigate the presence of central sensitization (CS) and/or NP in patients with RA.

**Methods:**

This descriptive, cross-sectional study used convenience sampling and included adult patients (≥ 18 years) with RA diagnosed according to the 2010 classification criteria of the American College of Rheumatology – ACR. The following instruments were applied: Central Sensitization Inventory (CSI), PainDETECT Questionnaire (PD-Q), and the 2010/2011 ACR criteria for fibromyalgia (FM), revised in 2016 (the latter applied only to patients with CSI ≥ 40). Descriptive and predictive analyses were conducted with a significance level of 5%.

**Results:**

A total of 113 patients were included (mean age 54.3 years; 93.81% female; mean disease duration 12.11 years). CS was identified in 59.29% of patients; among them, 47.76% met FM criteria, while 52.24% did not. Among patients with CS, 53.73% reported severe pain (Numeric Rating Scale, NRS), and 44.78% had high disease activity (Clinical Disease Activity Index, CDAI). CSI was a significant predictor of both NRS and CDAI in univariate (*p* < 0.001) and multivariate analyses (*p* = 0.037 and *p* = 0.009, respectively). According to PD-Q, 17.70% of patients had probable NP, and PD-Q scores were predictive of CDAI, NRS, and CSI (*p* < 0.001). Among patients with CS, 26.87% also showed overlapping NP.

**Conclusions:**

A substantial proportion of RA patients presented with CS-related pain, even in the absence of FM, suggesting a mixed (“top-down and bottom-up”) nociplastic pain profile. Both CS and NP were associated with higher pain levels and increased disease activity. This is the first Brazilian study to characterize the coexistence of CS-related pain (with or without FM) and NP in RA, offering relevant insights for the clinical management of chronic pain in this population. These findings reinforce that routine screening and assessment of CS and NP should be an integral and continuous component of RA management, preceding therapeutic adjustments and extending throughout follow-up.

## Introduction

Chronic pain (CP), defined as pain lasting ≥ 3 months, is a leading cause of disability worldwide, contributing to significant socioeconomic burdens and reduced quality of life [1, 2]. Pain conditions are generally classified into three main phenotypes: nociceptive, neuropathic, and nociplastic. Nociceptive pain arises from the activation of peripheral nociceptors in response to actual or potential tissue damage, with pain intensity proportional to the stimulus [3, 4]. Neuropathic pain (NP) is caused by a lesion or disease of the somatosensory nervous system and is typically characterized by a distribution corresponding to the affected neural structures. Nociplastic pain is primarily attributed to central sensitization (CS), a phenomenon in which pain arises from altered central pain processing, resulting in hypersensitivity to subthreshold or non-noxious stimuli. Allodynia and hyperalgesia are hallmark features of CS-related pain [3–5].

Rheumatoid arthritis (RA) is a chronic autoimmune disease that affects approximately 0.5–1% of the global population, with a prevalence of 0.46% in Brazil—higher than the South American average of 0.30%. RA predominantly affects women and typically manifests in the fifth decade of life, especially in developed countries. The disease is characterized by persistent synovial inflammation, leading to joint destruction, cartilage degradation, bone erosion, and in advanced cases, ankylosis. These pathological changes are driven by an imbalance in pro- and anti-inflammatory cytokines and increased activity of fibroblast-like synoviocytes, chondrocyte catabolism, and osteoclastogenesis [6–9]. Seropositivity for rheumatoid factor (RF) and/or anti-cyclic citrullinated peptide (anti-CCP) antibodies is associated with more aggressive disease.

Current therapeutic strategies aim to achieve remission or low disease activity, primarily through early diagnosis and timely use of disease-modifying antirheumatic drugs (DMARDs), including synthetic, biologic, and targeted agents [9–11]. Despite clinical remission or low disease activity, many patients continue to experience significant residual pain, which is not always explained by active inflammation. This phenomenon highlights the possible involvement of alternative pain mechanisms, particularly NP and CS.

Traditionally, pain in RA has been primarily attributed to peripheral nociceptive mechanisms. However, even with adequate therapy and in the absence of inflammation, many patients continue to experience significant residual pain. This suggests the involvement of other CP mechanisms, such as NP and/or pain related to CS. The latter is widely observed in patients with fibromyalgia (FM), although it may also occur in other diseases. Persistent pain, combined with easy access to anti-inflammatory drugs and analgesics, increases the risk of chronic and/or indiscriminate use of these medications, which can have a significant negative impact on patients’ quality of life [6, 12].

Nonetheless, these mechanisms often remain underdiagnosed, despite their established association with greater pain intensity, poorer quality of life, and increased functional impairment [11]. While some studies have investigated the isolated presence of CS, NP, or FM in patients with RA, few have explored the integrated overlap and interaction among these mechanisms within the same population, as well as their clinical impact in the disease context. This gap represents a significant challenge to the comprehensive understanding of pain in RA.

Furthermore, the presence of non-inflammatory pain mechanisms can significantly influence disease activity assessment. Widely used clinical tools, such as the Clinical Disease Activity Index (CDAI) and Disease Activity Score 28 (DAS-28), incorporate subjective components related to pain and patient global assessment. Thus, the presence of CS, NP, or FM can lead to artificially elevated scores even in the absence of active inflammation. This discrepancy may result in inappropriate therapeutic decisions, especially within the treat-to-target strategy that relies directly on these scores [6, 9, 11].

Given the need to identify the various mechanisms associated with CP, specific assessment instruments have been developed. These tools assist in identifying and quantifying CS, such as the Central Sensitization Inventory (CSI), validated for Portuguese-speaking populations [5]. There are also several tools for detecting NP, including the PainDETECT Questionnaire (PD-Q), which is easy to apply during medical consultations and also validated in Portuguese [13].

In this context, understanding the frequency, overlap, and impact of these mechanisms is essential for developing more individualized and effective diagnostic and therapeutic approaches in the management of RA, aiming to reduce adverse outcomes, inappropriate medication use, altered disease activity indices, and treatment discontinuation. This study aimed to evaluate the presence of CS and/or NP in patients with RA followed at a rheumatology outpatient clinic of a tertiary hospital in Northeast Brazil.

## Methods

A quantitative, descriptive, observational, cross-sectional study was conducted with patients followed at the rheumatology outpatient clinic of the Lauro Wanderley University Hospital (HULW), located in the city of João Pessoa, state of Paraíba, Brazil.

The target population consisted of individuals diagnosed with RA, regardless of race, sex, religion, or socioeconomic status, followed at the RA outpatient clinic of the aforementioned hospital between October 2023 and May 2024. The sample was selected using a non-probabilistic method, specifically convenience sampling, aiming to include the largest possible number of participants. Each patient was evaluated only once, regardless of the number of visits during the data collection period.

Patients previously classified as having RA according to the 2010 classification criteria of the American College of Rheumatology (ACR) and aged 18 years or older were included. Patients were excluded if they had cognitive impairments preventing them from understanding and responding to the study questionnaires; severe neurological comorbidities (e.g., stroke sequelae and degenerative neuropathies); psychiatric disorders with somatic symptoms of psychogenic origin (e.g., Somatic Symptom Disorder and Conversion Disorder); or a diagnosis of diabetes, regardless of disease duration.

Sociodemographic data, information on pain and/or comorbidities, and treatments were collected using a questionnaire specifically developed for this study and administered by the interviewer. To identify CS and NP, the CSI and the PD-Q were applied, respectively. The preliminary criteria for the diagnosis of FM (ACR 2010/2011 revised in 2016), were applied to participants with CSI scores ≥ 40. These questionnaires were completed independently by the participants after an initial explanation provided by the researcher on how to fill them out. The researcher remained available throughout the process to address any questions that might arise.

The CSI is divided into two parts. The first part consists of 25 questions related to the patient’s life experiences and symptomatology; the second part identifies possible previously diagnosed comorbidities. A CSI score ≥ 40 indicates positivity for CS, and patients can be classified according to score as moderate (40–49 points), severe (50–59 points), or extreme (≥ 60 points), with higher scores indicating greater sensitization [14]. The use of a cutoff point of 40 on the CSI is based on evidence that demonstrates its clinical validity in identifying patients with characteristics consistent with CS. Higher CSI scores are associated with increased self-reported symptomatology, and the cutoff of ≥ 40 has shown acceptable psychometric properties for this purpose [5;14].

The preliminary criteria for FM diagnosis consist of two sections. The first (Widespread Pain Index – WPI) quantifies painful regions by assigning 1 point for each region with pain in the last 7 days, totaling up to 19 points. The second section is the Symptom Severity Scale (SS), which quantifies the presence and intensity of other symptoms, with a maximum possible score of 12 points. A patient is considered positive for FM if they score WPI ≥ 7 and SS ≥ 5, or WPI between 4 and 6 and SS ≥ 9 [15].

The PD-Q is divided into four domains: pain intensity, pain course pattern, main pain areas, and sensory descriptors. For each question, six possible answers are provided, with scores ranging from zero (never) to five (very strongly). The total score ranges from − 1 to 38, and scores >18 are considered indicative of probable NP [13, 16].

To assess pain intensity, the Numeric Rating Scale (NRS) was used, with the following interpretations: 0 points – no pain; 1–3 points – mild pain; 4–6 points – moderate pain; 7–10 points – severe pain [16]. Disease activity was assessed using the CDAI, which combines physician and patient global assessments, tender and swollen joint counts, with thresholds for remission (≤ 2.8), low (>2.8–10), moderate (>10–22), and high disease activity (>22).

Patients were identified prior to their medical appointments by applying the inclusion and exclusion criteria and were then invited to participate in the study. Those selected agreed to participate by signing the informed consent form (ICF). Confidentiality and anonymity of all data collected were strictly maintained, and participation in the study did not interfere with their medical care. Additionally, all participants received thorough explanations about the study and had their questions answered.

After signing the ICF, the CSI and PD-Q were applied. After calculating the CSI scores, the FM diagnostic criteria were applied to those scoring ≥ 40 points. During the consultation, additional sociodemographic and disease-related data were collected, including the CDAI.

The study was approved by the Research Ethics Committee of the Center for Medical Sciences at UFPB, in accordance with Resolution CNS no. 466/2012 of the Brazilian Ministry of Health, which governs research involving human subjects in Brazil, under approval number CAAE 70626623.6.0000.5183.

Statistical analysis was conducted using numerical variables described by mean and standard deviation, and categorical variables described by absolute and relative frequency. The Shapiro–Wilk test was used to assess the normality of numerical variables. Pearson’s correlation coefficient was applied to evaluate correlations between normally distributed numerical variables. The Student’s t-test was used to compare means between two groups with normal distribution, while the Kruskal–Wallis test was applied for comparisons involving more than two groups or when the assumption of normality was not met. Univariate and multivariate linear regression models were used to explore associations between numerical variables, with adjustments for potential confounders when indicated. The significance level adopted was 5% (95% CI). All analyses were performed using R software, version 4.2.1.

## Results

A total of 162 patients were initially screened, of whom 113 were included in the study and 49 excluded. The main reason for exclusion was diabetes (36 patients), predefined due to its potential confounding effects on pain mechanisms, including diabetic neuropathy and metabolic inflammation. Additional exclusions were due to neurological disorders, such as significant sequelae, wheelchair dependence, or Alzheimer’s disease (6 patients), and psychiatric disorders, including bipolar disorder, severe active depression, or schizophrenia (5 patients). Two patients withdrew before study completion. Notably, the exclusion of patients with psychiatric comorbidities had minimal impact on the final sample, indicating that diabetes was the predominant factor influencing cohort size.

A total of 113 patients with RA were included in the study. Their sociodemographic and clinical characteristics are summarized in Table 1. The sample was predominantly female (93.81%), with a mean age of 54.3 years (range: 25–77). Most participants self-identified as mixed-race (75.22%), and 57.52% were in a conjugal relationship. Regarding educational attainment, 43.36% had completed only primary education, and 41.59% had completed secondary education. The majority (69.02%) resided in the capital or metropolitan area.

Regular physical activity was reported by 24.78% of participants, and tobacco use was rare (3.54%). The most prevalent comorbidities were systemic arterial hypertension (SAH) (43.36%), osteoarthritis (30.97%), and a previous history of Chikungunya infection (10.62%).

Table 2 presents the types of pain reported by patients, disease activity levels, pain intensity as measured by the NRS, and the presence of FM criteria among patients with positive CSI scores, based on the instruments applied. CS was observed in 59.29% of the sample. Of these, only 47.76% met the criteria for FM, while 52.24% had CS without meeting FM criteria. Regarding NP, 17.70% of the sample was classified as probable NP; 42.48% of participants reported severe pain at the time of evaluation, and 43.36% of the total sample were in disease remission or had low disease activity (CDAI).

**Table 1:** General profile, clinical profile and treatment characteristics of patients with rheumatoid arthritis

Variables	Value	Variables	Value
**Gender – ** ***n*** ** (%)**		**Physical activity – ** ***n*** ** (%)**	
Female	106 (93.81)	Positive	28 (24.78)
**Age (in years)**		**RA diagnosis (in years)**	
Min - Max	25–77	Min – Max	1–35
Avg. ± Std. dev.	54.3 ± 12.22	Avg. ± Std. dev.	12.11 ± 6.97
**Race – n (%)**		**RF – n (%)**	
White	23 (20.35)	Positive	64 (56.64)
Brown	85 (75.22)	Negative	42 (37.17)
Black	4 (3.54)	Unknown	7 (6.19)
Other	1 (0.88)		
		**Anti-CCP – n (%)**	
**Marital Status – n (%)**		Positive	40 (35.40)
Married or cohabiting	65 (57.52)	Negative	39 (34.51)
Single	26 (23.01)	Unknown	34 (30.09)
Widowed	13 (11.50)	RF and ANTI-CCP Positive	26 (23.01)
Divorced	9 (7.96)		
		**Medications**	
**Education Level – n (%)**		**sDMARD – n (%)**	
Elementary*	49 (43.36)	MTX	37 (32.74)
Secondary*	47 (41.59)	Other sDMARD	53 (46.90)
Post-Secondary*	17 (15.04)	MTX + other sDMARD	9 (7.96)
**Residence – n (%)**		**bDMARD – n (%)**	
Capital or metropolitan	78 (69.02)	Non-TNF inhibitors	22(19.47)
		Anti-TNF agents	12(10.62)
**Comorbidities – n (%)**			
Hypertension	49 (43.36)	**tDMARD – n (%)**	
Osteoarthritis	43 (38.05)	UPA, BARI or TOFA	17(15.04)
Chikungunya (previous)	12 (10.62)		
		**Other Medications – n (%)**	
**Smoking – n (%)**		Prednisone	28 (24.78)
Positive	4 (3.54)	Gabapentinoid	11 (9.73)
		SSRIs, SNRIs or Amitriptyline	12 (10.62)
		Gabapentinoid + SSRI/SNRI	9 (7.96)

*With Educational level completed or attended (incomplete). Min: Minimum. Max: Maximum. Avg.: Average (Mean). Std. dev.: Standard Deviation. RF: Rheumatoid Factor. Anti-CCP. Anti-Cyclic Citrullinated Peptide Antibodies. sDMARDs: Synthetic Disease-Modifying Anti-Rheumatic Drugs. MTX: Methotrexate. bDMARDs: Biologic Disease-Modifying Anti-Rheumatic Drugs. TNF: Tumor Necrosis Factor; tDMARDs: Targeted Synthetic Disease-Modifying Anti-Rheumatic Drugs. UPA: Upadacitinib. BARI: Baricitinib. TOFA: Tofacitinib. SSRIs: Selective Serotonin Reuptake Inhibitors. SNRIs: Serotonin and Norepinephrine Reuptake Inhibitors

**Table 2:** Distribution of pain types and disease activity index in rheumatoid arthritis patients

Variables	Value *n* (%)	Mean ± standard deviation
**CSI**		43.40 ± 16.80
Negative (< 40)	46 (40.71)	
Moderate (40–49)	19 (16.81)	
Severe (50–59)	25 (22.12)	
Extreme (60–100)	23 (20.35)	
**Fibromyalgia Criteria**		
**Applied**	67 (100)	
FM Positive	32 (47.76)	
**PD-Q – n (%)**		11.90 ± 7.17
Unlikely (< 13)	64 (56.64)	
Uncertain (13–18)	29 (25.66)	
Probable (> 18)	20 (17.70)	
**Pain NRS**		5.15 ± 3.01
No pain (0)	18 (15.93)	
Mild (1–3)	15 (13.27)	
Moderate (4–6)	32 (28.32)	
Severe (7–10)	48 (42.48)	
**CDAI**		17.20 ± 14.90
≤ 2.8 (remission)	9 (7.96)	
> 2.8 and ≤ 10 (low)	40 (35.40)	
> 10 and ≤ 22 (moderate)	25 (22.12)	
> 22 (high)	35 (30.97)	
Not reported	4 (3.54)	

CSI: Central sensitization inventory; FM: Fibromyalgia. PD-Q: Pain detect questionnaire; NRS = Numeric rating scale; CDAI: Clinical Disease Activity Index; Avg.: Average (Mean). Std. dev.: Standard Deviation

When analyzing only the patients with CS (Table 3), a prevalence of high disease activity (CDAI > 22) was observed in 44.78% of cases, and 20.90% had moderate disease activity (CDAI > 10 and ≤ 22). Only 2.99% of patients with CS were in remission (CDAI ≤ 2.8). Additionally, pain scores revealed that 53.73% of patients were characterized as having severe pain (NRS between 7 and 10 points), while 17.91% reported no pain or mild pain (NRS ≤ 3 points). An overlap between CS and NP was identified in 18 individuals.

Patients with probable NP (PD-Q > 18) also showed high disease activity: 50% of them had CDAI scores > 22, and none were in remission or had low disease activity. A similar pattern was seen in pain levels, with 55% of NP patients reporting severe pain.

**Table 3:** Classification of pain intensity and disease activity in patients positive for central sensitization and/or probable neuropathic pain

Variables	CS Positive (CSI ≥ 40)	Probable NP (PD-Q > 18)	Without CS and without NP
**CDAI – ** ***n*** ** (%)**			
≤ 2.8 (remission)	2 (2.99)	00	7 (15.91)
> 2.8 and ≤ 10 (low)	18 (26.87)	5 (25.00)	22 (50.00)
> 10 and ≤ 22 (moderate)	14 (20.90)	5 (25.00)	10 (22.73)
> 22 (high)	30 (44.78)	10 (50.00)	4 (9.09)
Not reported	3 (4.48)	00	1 (2.27)
Avg. ± Std. dev.	21.84 ± 15.32	23.30 ± 15.60	10.29 ± 11.76
**Pain NRS at the moment – n (%)**			
No pain (0)	5 (7.46)	00	13 (29.55)
Mild (1–3)	7 (10.45)	00	8 (18.18)
Moderate (4–6)	19 (28.36)	9 (45.00)	13 (29.55)
Severe (7–10)	36 (53.73)	11 (55.00)	10 (22.73)
Avg. ± Std. dev	6 ± 2.63	7 ± 1.45	3.66 ± 3.06
**PD-Q – n (%)**			
Unlikely (< 13)	27 (40.30)	-	-
Uncertain (13–18)	22 (32.84)	-	-
Probable (> 18)	18 (26.87)	-	-
Avg. ± Std. dev	14.82 ± 6.85	**-**	-

CS: Central sensitization; CSI: Central sensitization inventory; NP: Neuropathic pain; PD-Q: Pain detect questionnaire; NRS = Numeric rating scale; CDAI: Clinical Disease Activity Index; Avg.: Average (Mean). Std. dev.: Standard Deviation

**Fig. 1:**
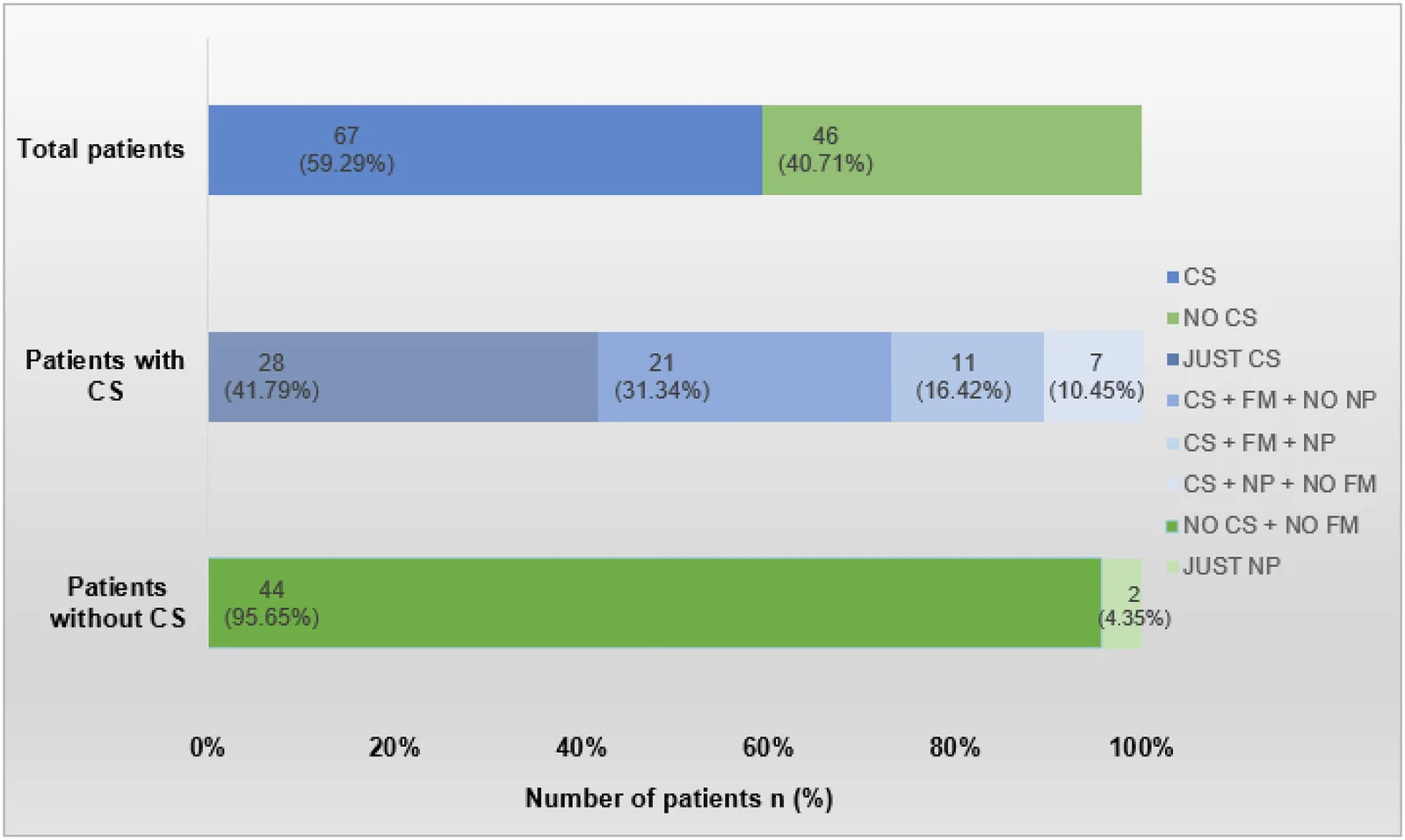
Overlap of central sensitization (CS), fibromyalgia (FM), and neuropathic pain (NP) among study participants. Groups include patients without CS and FM (NO CS + NO FM), those with only NP (JUST NP), those with CS only (CS), patients without CS (NO CS), those with CS and FM combined with NP (CS + FM + NP), CS and FM without NP (CS + FM + NO NP), patients with only CS (JUST CS), and those with CS and NP but without FM (CS + NP + NO FM)

The Fig. 1, illustrates the overlap among the identified pain mechanisms. Among participants with CS, 31.34% presented CS associated with FM without NP, whereas 16.42% exhibited a combination of CS, FM, and NP. Additionally, 10.45% had CS associated exclusively with NP, without FM. Notably, none of the participants without CS met the diagnostic criteria for FM.

**Fig. 2:**
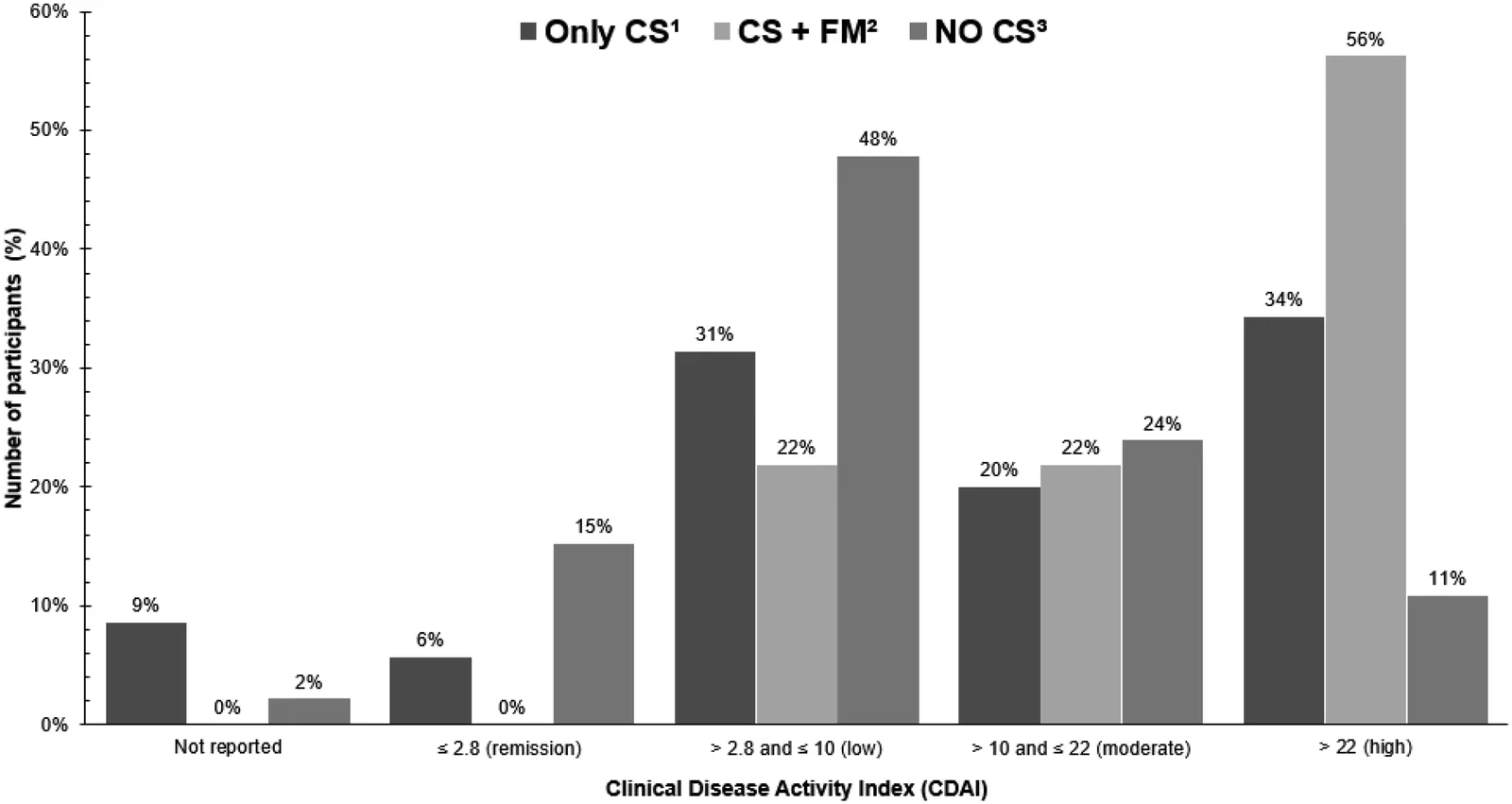
Comparison of disease activity between patients with central sensitization alone and those with fibromyalgia diagnostic criteria. *A global statistically significant difference was observed (Kruskal *p* < 0.001). (1) Only CS: Patients with central sensitization only (mean CDAI = 20.0; standard deviation = 17.0; var = 356.26). (2) CS + FM: Patients with central sensitization who also met diagnostic criteria for Fibromyalgia (mean CDAI = 23.74; standard deviation = 13.47; var = 148.95). (3) No CS: Patients without central sensitization (mean CDAI = 10.72; standard deviation = 11.70; var = 136.88)

Patients positive for CS were also assessed for FM diagnosis, as well as NRS and CDAI scores, and compared to patients without CS. It was observed that patients with FM had a mean CDAI score similar to those with CS but without FM, with mean of 23.74 and 20 points, respectively. However, 78% of patients with FM had moderate and/or high disease activity according to CDAI, and none were in remission. In contrast, 54% of patients without FM met criteria for moderate and/or high disease activity, and 6% were in remission (Fig. 2). On the other hand, the majority of patients without CS (48%) presented with low disease activity, and their mean CDAI score was 10.72, lower than those observed in the other groups.

**Fig. 3:**
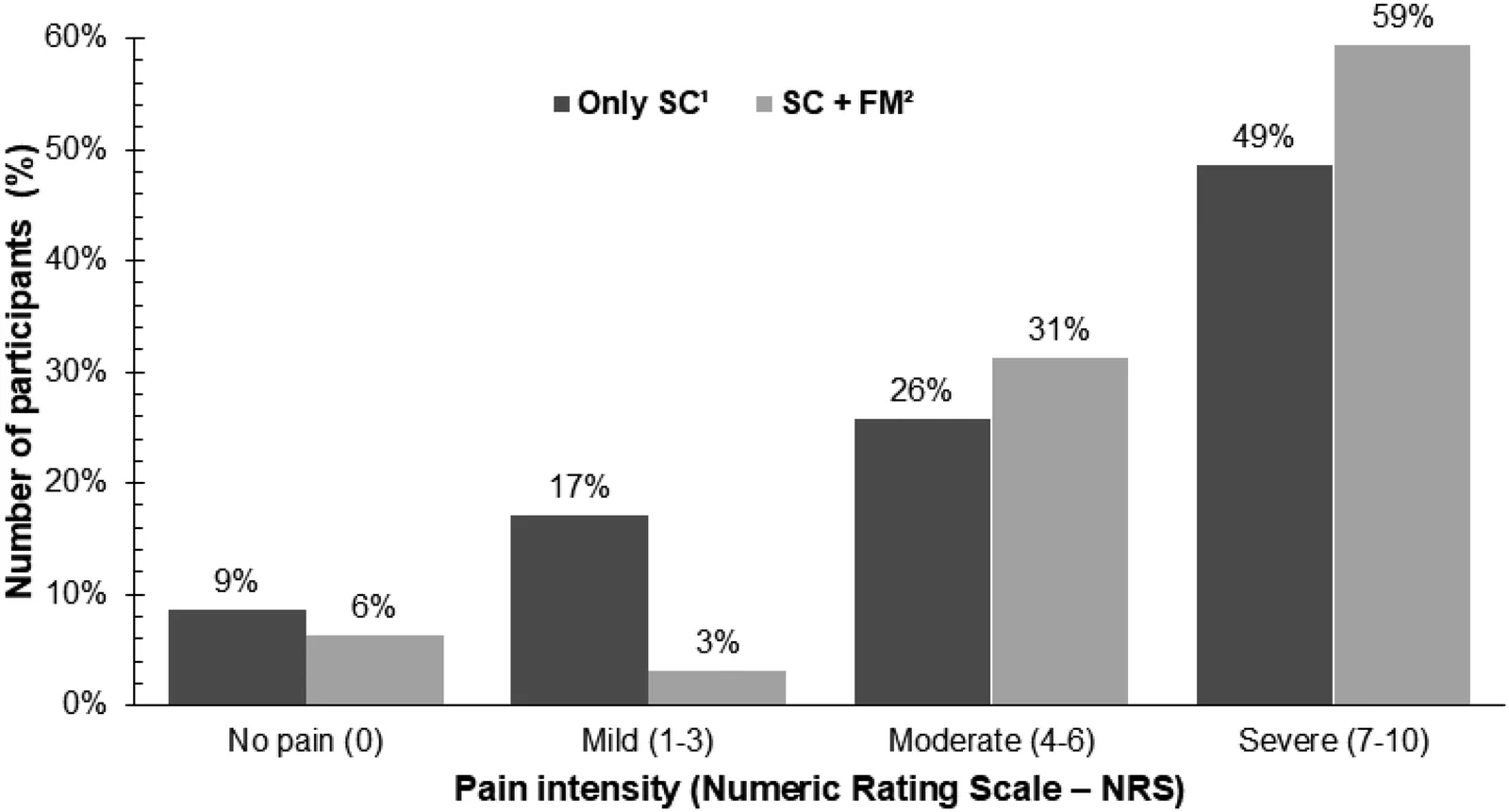
Pain score in patients with central sensitization, with or without Fibromyalgia diagnostic criteria. * A global statistically significant difference was observed (Kruskal *p* < 0.001). (1) Only CS: Patients with central sensitization only (mean Numeric Rating Scale = 6.50; standard deviation = 2.78; var = 6.83). (2) CS + FM: Patients with central sensitization who also met diagnostic criteria for Fibromyalgia (mean Numeric Rating Scale = 7; standard deviation = 2.37; var = 4.59). (3) No CS: Patients without central sensitization (mean Numeric Rating Scale = 3.83; standard deviation = 3.09; var 9.57)

Regarding pain intensity, the mean scores were very similar among patients with CS: 7 points in the FM group and 6.5 in the group without FM. However, as observed for CDAI, the NO CS group reported lower pain intensity, with a mean NRS score of 3.83 (Fig. 3).

There were statistically significant overall differences among groups for both pain and CDAI scores (*p* < 0.001 for both). In pairwise comparisons, the “CS + FM” group showed significantly higher scores compared to the “NO CS” group, both for pain (*p* = 0.010) and for CDAI (*p* = 0.016). In contrast, no significant differences were found between the “CS + FM” and “Only CS” groups (pain, *p* = 0.736; CDAI, *p* = 1.000), nor between the “Only CS” and “NO CS” groups (pain, *p* = 0.422; CDAI, *p* = 0.118).

Correlation analysis (Table 4) showed positive associations between CSI and CDAI (*r* = 0.446, *p* < 0.001), CSI and NRS (*r* = 0.443, *p* < 0.001), and PD-Q with both NRS (*r* = 0.396, *p* < 0.001) and CDAI (*r* = 0.391, *p* < 0.001). PD-Q was also positively correlated with CSI (*r* = 0.359, *p* < 0.001).

Regression analyses (Table 4) indicated that CSI was a significant predictor of NRS (univariate *p* < 0.001; multivariate *p* = 0.037) and CDAI (univariate *p* < 0.001; multivariate *p* = 0.009). PD-Q was a predictor of NRS, CDAI, and CSI in univariate models (all *p* < 0.001). Neither smoking status nor regular physical activity was significantly associated with CSI, PD-Q, or disease activity.

**Table 4:** Univariate, multivariate, and correlation analyses in patients with rheumatoid arthritis

Analysis			
	*R*²	*p*-value	Effect size
**Univariate**			
PD-Q vs. CDAI	0.153	< 0.001	0.808
PD-Q vs. CSI	0.359	< 0.001	1.41
Smoking vs. PD-Q	0.0344	0.049	-
Exercise vs. PD-Q	0.0639	0.641	-
Smoking vs. CSI	1.36e-6	0.990	-
Exercise vs. CSI	0.0796	0.830	-
NRS¹ vs. CSI	0.196	< 0.001	0.0792
NRS¹ vs. PD-Q	0.157	< 0.001	0.166
CDAI vs. CSI	0.199	< 0.001	0.394
CDAI vs. Exercise	0.101	0.629	-
CDAI vs. Smoking	0.011	0.279	-
**Multivariate**			
NRS¹ vs. CSI vs.FM	0.106	0.037	0.076
CDAI vs. CSI vs. FM	0.122	0.009	0.568
**Correlations**	**Pearson’s r**	**p-value**	
Diagnosis² vs. CSI	-0.084	0.385	-
Diagnosis² vs. PD-Q	0.045	0.646	-
NRS¹ vs. PDQ	0.396	< 0.001	-
NRS¹ vs. CSI	0.443	< 0.001	-
PD-Q vs. CDAI	0.391	< 0.001	-
CSI vs. CDAI	0.446	< 0.001	-
**Correlations**	**T-Student**	**p-value**	
PD-Q vs. RF	0.737	0.463	-
PD-Q vs. ANTI-CCP	0.639	0.525	-
CSI vs. RF	0.855	0.395	-
CSI vs. ANTI-CCP	-0.230	0.818	-

1. NRS: Numeric Rating Scale, used to assess pain intensity; 2.Time to diagnosis; CSI: Central Sensitization Inventory. PD-Q: Pain detect questionnaire. CDAI: Clinical Disease Activity Index. RF: Rheumatoid Factor. Anti-CCP: Anti-Cyclic Citrullinated Peptide Antibody; FM: Fibromyalgia

## Discussion

A predominantly female sample (93.81%) was identified, with a mean age of approximately 54 years, as expected for RA patients in Brazil. This finding is corroborated by Sacilotto et al. [17], who reported a sample composed of 90% women with a mean age of 56.7 years. Although studies from other countries report slightly different samples, most also indicate a female predominance greater than 70% and a mean age between 50 and 60 years [18–20]. The mean duration of diagnosis (12.11 years) remained within the range observed in recent studies, demonstrating compatibility of the sample with the contemporary profile of RA.

In the present study, more than 50% of patients tested positive for RF and more than 30% for anti-CCP. The presence of these markers is associated with a more aggressive disease profile, which can hinder and prolong disease control. Rocha, Baldo, and Andrade [21] highlighted that patients positive for RF tend to have less effective clinical responses to TNF-α inhibitors but may respond better to B-cell depletion therapies, such as rituximab. Additionally, the presence of anti-CCP can interfere with the effectiveness of DMARDs, often requiring the early initiation of combination therapy [22]. This profile has the potential to lead to more challenging clinical management, as demonstrated in the present study’s sample.

Regarding pharmacological treatment, patients were undergoing appropriate therapy in accordance with recommendations from the main rheumatology societies, including Brazil’s Clinical Protocol and Therapeutic Guidelines for RA [23, 24]. More than 75% of the sample were on DMARDs, either as monotherapy or in combination. However, despite appropriate therapy, most patients continued to report high pain levels and high disease activity, based on NRS and CDAI scores. The persistence of pain despite adequate treatment reinforces the importance of investigating other pain mechanisms in this patient group [25]. One contributing factor often described in the literature is the coexistence of FM in RA patients [22], present in an average of 21% of cases, ranging from 4.9% to 52.4% according to the meta-analysis by Duffield et al. [26].

The mechanism behind the development of nociplastic pain in RA is not yet fully understood. Clauw [22] suggests that CS in RA patients results from the chronic inflammation characteristic of the disease, lowering the pain threshold and amplifying pain perception even in the absence of noxious stimuli. This may be exacerbated by dysfunction in the descending inhibitory pathways, which normally modulate and reduce pain, leading to CS associated with the primary disease [22]. In turn, Meert et al. [27] identified that CS may result from two distinct mechanisms: “top-down” (from the brain to the periphery) or “bottom-up” (from the periphery to the brain). In RA, the inflammatory stimulus could result in prolonged hyperactivity of dorsal horn neurons, contributing to an exaggerated pain response and leading to predominantly “bottom-up” CS. Both authors propose that top-down and bottom-up mechanisms may coexist and overlap [22, 27].

The prevalence of CS among RA patients in our sample was nearly 60%, a higher percentage than even the elevated rates reported by Mesci et al. [28] 48.3% and Guler, Celik, and Ayhan [29] 41.10%. Adami et al. [11] and Salaffi et al. [31] reported lower rates of CS in this group, 29% and 36.5%, respectively, reinforcing the association between RA and CS and the need to better understand this phenomenon and its impact on the clinical profile. Results from our study showed that just over 30% of patients presented with CS without FM, demonstrating that RA can evolve with CS-related pain in a significant portion of the sample, even without meeting FM criteria.

Considering the neurophysiology of pain, anxiety and depression may contribute to the development of a more primary form of nociplastic pain represented by FM or a secondary presentation, as seen in CS without FM [26], or even a mixed CS profile (“top-down and bottom-up”) in RA [27]. From this perspective, the high prevalence may be justified. Furthermore, the presence of CS may explain discrepancies between physician and patient assessments regarding clinical improvement, pain levels, and disease perception, as well as the frequent occurrence of residual pain in RA patients [25, 30–32].

The study by Mesci et al. [28] found a positive association between pain levels and disease activity patients with CS tend to have higher levels of pain and higher CDAI values which corroborates the results of our study. In the presence of CS, greater pain reactivity is expected. The study by Adami et al. [11], which evaluated the association between CSI, disease activity, and functional disability, also identified a relationship between pain intensity and disease activity via CSI scores. It found that the association between CSI scores and disease activity was mainly mediated by persistent inflammation and was not the result of faulty global assessment or subjective pain intensity reported on the NRS. The authors concluded that CS in RA significantly impacts functional disability.

The study by Saitou et al. [20] also found an association between CSI and disease activity using different disease activity metrics (CDAI, Simplified Disease Activity Index – SDAI, and DAS-28. However, Guler et al. [29] did not identify any relationship between CSI and pain level or disease activity (DAS-28). The use of a single metric that incorporates an inflammatory marker may have been a confounding factor, as CS can occur even in the absence of inflammation—especially when nociplastic pain is primary.

Sarzi-Puttini et al. [25] found that patients with residual pain are frequently considered to have treatment failure, leading to medication overuse or increased polypharmacy, as well as increased treatment costs and adverse effects. Since pain amplification caused by RA may be responsible for a portion of residual pain symptoms, caution is necessary regarding the excessive use of analgesics, corticosteroids, NSAIDs, and DMARDs.

The results of this study showed that patients with CS predominantly reported severe pain and moderate and/or high disease activity, even when under appropriate pharmacological treatment. If residual pain and disease activity are being driven by an undiagnosed CS, the use of anti-inflammatory agents and DMARDs alone may not sufficiently control pain or induce remission and/or low disease activity. In this regard, Clauw [22] suggests the inclusion of interventions that directly modulate the CNS, as a complement to traditional inflammation-focused approaches.

In this context, the use of tricyclic antidepressants and SNRIs is recommended to restore the function of descending inhibitory pathways, along with medications that modulate calcium and/or sodium influx, which may be effective in reducing neuronal hyperexcitability [25, 27, 31]. Salaffi et al. [33] demonstrated that Janus Kinase (JAK) inhibitors appear to have a positive effect on pain-related variables, particularly central sensitization and pain catastrophizing, which may originate from extra-synovial mechanisms.

The aforementioned studies did not find any association between disease duration and CS, which aligns with our study. However, this is a surprising result, given that longer exposure to nociceptive damage would theoretically have the potential to trigger CS, as suggested in the literature. Likewise, no correlation was found between autoantibody markers (RF and anti-CCP), despite their association with more severe disease presentations. Smoking and physical activity also did not appear to influence the additional pain mechanisms identified, although this result may have been affected by the low frequency of these variables in the sample. Other studies did not evaluate smoking status or physical activity levels.

The presence of NP in chronic inflammatory diseases has gained increasing attention in recent years, due to its potential impact on quality of life and its interference with treatment efficacy. Just over 17% of this study’s sample presented with probable NP, according to the PD-Q. These values are consistent with those reported in the literature, which range between 12.5% and 40%, depending on the instrument used [34–38].

Di Carlo, Smerilli, and Salaffi [39] argued that NP in RA results from a complex combination of factors. It may arise due to compressive mechanisms triggered when chronic joint inflammation alters local or even neuronal morphology, leading to nerve compression by adjacent structures. This is seen in some presentations of carpal tunnel syndrome when triggered by RA.

Another possibility is that NP may also develop due to the activation of glial cells in the central nervous system (CNS), particularly in the spinal cord. These cells, when activated by pro-inflammatory cytokines released during RA such as IL-1β, IL-6, and TNF-alpha—produce mediators that amplify NP through neuronal damage and continued activation of glial cells. This neurosensitization process can occur from the early stages of RA, suggesting that NP is not merely a consequence of prolonged inflammation but may also be an early feature of the disease [39].

The presence of synovial inflammation can lead to perineuritis, an inflammation of peripheral nerves, as observed in the palmar digital nerves of RA patients. This theory of peripheral nerve damage is supported by the study by Pereira, Lourenço, and Assis [37], who assessed the presence of neuropathies in RA patients and found that 48.5% had some form of peripheral neuropathy (either axonal or demyelinating), confirmed by electroneuromyography. Sousa et al. [40] identified axonal damage caused by cytokines, antibodies, or cytotoxic cells as likely causes of NP in rheumatologic disorders.

The findings of this study indicated a positive correlation and predictive value between NP (as defined by probable PD-Q), disease activity, and pain intensity. According to these results, patients with probable NP tend to report higher pain levels and greater disease activity scores, similarly to what is observed in CS. Similar findings were reported by Koop et al. [35], who found that RA patients with NP had higher pain levels, higher DAS-28 scores, poorer quality of life, and a higher likelihood of disability. In turn, Garip et al. [34] identified an association between NP and pain intensity but did not find a relationship between NP and the number of painful joints. Conversely, Radwan and Borai [38] found an association between pain intensity and the number of painful joints, and the latter also identified a relationship between disease duration and NP levels unlike the findings in this study.

PD-Q was a predictor of CS, with a proportion of patients exhibiting overlapping NP and CS. Similar results were observed in other studies [19, 20]. Some authors argue that NP may be a symptom of CS, that tools designed to identify NP may be useful for identifying CS, or that the co-occurrence of NP and CS may be explained by shared mechanisms of pain development. However, this relationship is not yet well-established in the literature, and further studies are needed to identify and analyze these associations, their impacts, and when NP functions as a symptom of CS and/or as a distinct primary or secondary pain mechanism [19, 20].

The differences found among studies investigating NP in RA may be influenced by racial variations and sample size, since these are generally small studies, as well as differences in pain management strategies used in different populations, the inclusion or exclusion of patients with comorbidities that may cause NP, such as diabetes, and the use of different assessment tools (e.g., PD-Q vs. DN4), as suggested in several studies [20, 35, 38].

The early identification and treatment of mechanisms associated with NP development are essential for effective pain management in RA patients and for achieving disease remission, just as in the presence of CS in RA patients. Effective pain management requires strategies that address both central modulation and peripheral inflammatory mechanisms. A comprehensive therapeutic approach including both pharmacological and non-pharmacological components, and the use of different drug classes to control nociception, CS, and NP is necessary to mitigate the harmful potential of the various CP mechanisms associated with RA.

The results of this study reinforce that pain reported by patients with RA may, in many cases, be dissociated from the objective inflammatory activity of the disease, being instead related to CP mechanisms. This dissociation is particularly relevant in the clinical setting, considering that widely used tools to guide therapeutic decisions such as the CDAI and DAS-28 include subjective components directly influenced by the patient’s pain perception, such as global assessment and tender joint count. Thus, the presence of CS and/or NP may result in artificially elevated scores, even in the absence of active inflammation, raising important concerns about the accuracy of these indices in reflecting the true inflammatory activity of the disease [9, 41].

This distortion has relevant clinical implications, especially in the context of the treat-to-target strategy, which is widely recommended and adopted in modern rheumatology practice to achieve remission or low disease activity [41]. As this strategy relies directly on clinical scores to guide therapeutic decisions, the influence of non-inflammatory pain mechanisms may lead to unnecessary intensification of immunosuppressive treatment, increasing the risk of adverse effects and healthcare costs. Therefore, it is crucial to consider these mechanisms when interpreting RA activity scores, incorporating specific tools to detect CS and NP, as well as promoting the development of new assessment instruments that more comprehensively evaluate disease activity in RA, including the presence of these other CP mechanisms. This integrated approach may support more individualized, safe, and effective clinical decisions, ensuring adequate pain control without compromising the accuracy of inflammatory disease activity assessment.

Considering its relevance in the context of RA, CP treatment cannot be disregarded. In the present sample, the low use of antidepressants and gabapentinoids may reflect the underdiagnosis of CS and NP, as well as other contributing factors such as financial constraints, limited access to specialized care, tolerability issues, and concerns about side effects. Although treatment was briefly mentioned, a detailed discussion of all therapies used or not used was beyond the scope and methodological design of this study. Given the breadth, complexity, and amount of data required for a thorough analysis of pharmacological management and its implications for RA, CS, and NP, we believe this topic deserves dedicated future investigations.

This study presented some limitations that should be recognized. The limited timeframe available for data collection and study implementation restricted the sample size. In addition, the scarcity of previous studies on this topic limited the possibility of in-depth comparisons and made it difficult to contextualize findings within the existing body of knowledge. Other limitations inherent to studies of this nature include self-reporting bias, participants’ difficulties in completing or bringing requested tests to consultations, and methodological limitations related to the study design, which may impact the generalizability of the results. Despite these constraints, the findings contribute significantly to the understanding of the topic and may serve as a foundation for future investigations.

In contrast, an important strength of this study is the careful exclusion of patient groups that could act as major confounders in the interpretation of pain mechanisms, such as individuals with diabetes, psychiatric disorders, or significant neurological conditions. By applying these criteria, the final sample was composed of patients less influenced by overlapping conditions that themselves may generate or amplify CP. This methodological choice allowed for a clearer characterization of CS and NP specifically within the context of RA, reducing bias and increasing the internal validity of the findings.

## Conclusions

This is the first Brazilian study to identify and analyze the coexistence of CS-related pain (with or without FM) and/or NP in RA, offering important contributions to the management of CP in this disease. CS was observed at significant rates, even in the absence of FM, suggesting a mixed presentation of nociplastic pain (“top-down and bottom-up”) in RA. It was demonstrated that patients with CS have higher levels of pain and disease activity, and that those with moderate and/or high disease activity, or higher scores on the pain scale, are more likely to test positive for CS. A smaller proportion of patients presented with NP, which was also associated with higher CDAI scores and increased pain intensity.

Building on the present findings, routine screening and assessment of CS and NP should be an integral and continuous component of RA management, not only preceding therapeutic adjustments but also continuing throughout follow-up. Moreover, further research is warranted to elucidate these associations, clarify their clinical relevance, and guide the development of tailored treatment approaches. In particular, future longitudinal studies are needed to determine whether actively managing CS and NP can modify RA outcomes.

## Data Availability

The datasets used and/or analyzed during the current study are available from the corresponding author on reasonable request.

## Abbreviations

Anti-CCP: Anti-Cyclic Citrullinated Peptide Antibody
Anti-TNF: Anti-Tumor Necrosis Factor
BARI: Baricitinib
bDMARDs: Biologic Disease-Modifying Anti-Rheumatic Drugs
CDAI: Clinical Disease Activity Index
CP: Chronic Pain
CS: Central Sensitization
CSI: Central Sensitization Inventory
DMARD: Disease-Modifying Antirheumatic Drugs
FM: Fibromyalgia
GABA: Gamma-Aminobutyric Acid
JAK: Janus Kinase Inhibitors
MTX: Methotrexate
NP: Neuropathic Pain
NRS: Numeric Rating Scale
RA: Rheumatoid Arthritis
RF: Rheumatoid Factor
sDMARDs: Synthetic Disease-Modifying Antirheumatic Drugs
SNRIs: Serotonin-Norepinephrine Reuptake Inhibitors
SS: Symptom Severity Scale
SSRI: Selective Serotonin Reuptake Inhibitor
tDMARDs: Targeted Synthetic Disease-Modifying Anti-Rheumatic Drugs
TOFA: Tofacitinib
UPA: Upadacitinib
WPI: Widespread Pain Index
